# Kv7 Channels Can Function without Constitutive Calmodulin Tethering

**DOI:** 10.1371/journal.pone.0025508

**Published:** 2011-09-28

**Authors:** Juan Camilo Gómez-Posada, Paloma Aivar, Araitz Alberdi, Alessandro Alaimo, Ainhoa Etxeberría, Juncal Fernández-Orth, Teresa Zamalloa, Meritxell Roura-Ferrer, Patricia Villace, Pilar Areso, Oscar Casis, Alvaro Villarroel

**Affiliations:** 1 Unidad de Biofísica, Consejo Superior de Investigaciones Científicas-Universidad del País Vasco/Euskal Herriko Unibersitatea, Leioa, Spain; 2 Dept. Farmacología, Universidad del País Vasco, Leioa, Spain; 3 Dept. Fisiología, Universidad del País Vasco, Leioa, Spain; Sackler Medical School-Tel Aviv University, Israel

## Abstract

M-channels are voltage-gated potassium channels composed of Kv7.2-7.5 subunits that serve as important regulators of neuronal excitability. Calmodulin binding is required for Kv7 channel function and mutations in Kv7.2 that disrupt calmodulin binding cause Benign Familial Neonatal Convulsions (BFNC), a dominantly inherited human epilepsy. On the basis that Kv7.2 mutants deficient in calmodulin binding are not functional, calmodulin has been defined as an auxiliary subunit of Kv7 channels. However, we have identified a presumably phosphomimetic mutation S511D that permits calmodulin-independent function. Thus, our data reveal that constitutive tethering of calmodulin is not required for Kv7 channel function.

## Introduction

Neuronal Kv7.2 and Kv7.3 subunits (encoded by the KCNQ2 and KCNQ3 genes) are the main components of the tetrameric voltage-dependent K^+^ M-channel that operates in the subthreshold voltage range of action potential generation. Mutations that cause a 25% reduction in current provoke Benign Familial Neonatal Convulsions (BFNC) [1], a dominantly inherited idiopathic human epilepsy [2], indicating that it is essential to attain a threshold of M-channel function to maintain normal physiological activity.

Of the five members of the Kv7 family, Kv7.4 and Kv7.5 can also contribute to M-channels [2]. Sequence analysis predicts the presence of four helical regions (A-D) in the intracellular C-terminus [3], which is the main site for interactions with other proteins [4]. Calmodulin (CaM) binds to helices A and B [3] and gates Ca^2+^-dependent and Ca^2+^-independent inhibition of M-channels [5]–[8]. Blockage by N-ethylmaleimide (NEM) of Ca^2+^-CaM mediated inhibition of the M-current in sympathetic neurons points towards a main role of helix B for this regulatory activity [7].

Similar to SK potassium channels [9], CaM has been considered to be an integral part of the M-channel complex [10]; [11]. In fact, it has been proposed that CaM modulates some steps in channel biosynthesis such as folding or assembly [10]; [12]; [13]. However, it still remains unclear whether CaM binding to Kv7 channels is constitutive and required for channel function [7]; [10]; [14]. A thorough understanding of the role of CaM binding in Kv7 channels function is not yet available, partly because CaM binding deficient mutants are not functional [10]; [14]; [15]. We have previously investigated the impact of several mutations located in helix A, revealing a strong correlation between CaM binding and Kv7.2 surface expression. Interestingly, some mutations linked to BFNC are located in helix A [16]; [17] and reduce CaM binding, thereby leading to ER retention [14]; [15]. We decided to study the impact of a mutation located in helix B that disrupt CaM binding [3]. We found unexpectedly that this mutant can traffic to the surface and is functional. This finding suggest that Kv7.2 can assemble and function constitutively binding to CaM, indicating that CaM is not an integral part of the M-channel complex.

## Results

**Figure 1:**
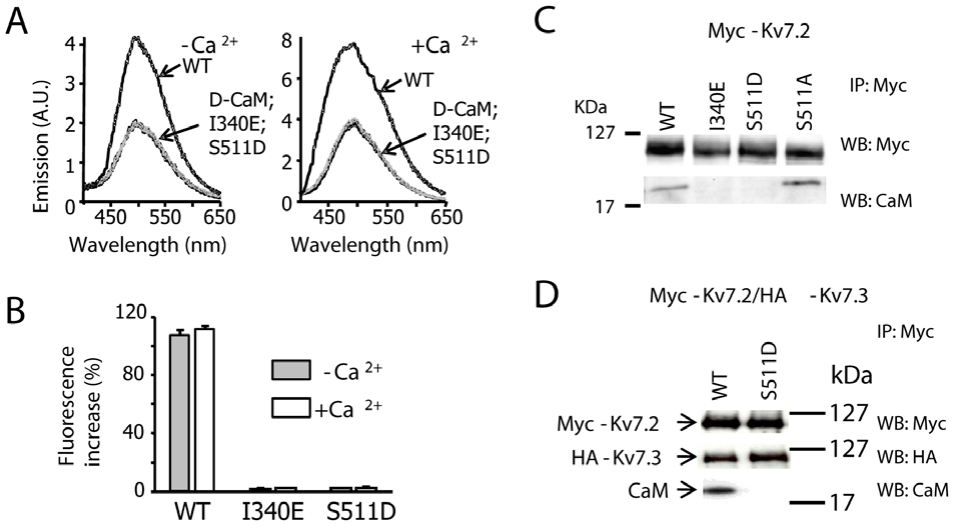
The S511D and I340E mutations disrupt the CaM interaction. ***A.*** Emission spectra of 12.5 nM D-CaM in the absence (left panel; 10 mM EGTA and no added Ca^2+^), and in the presence of 2.0 µM free Ca^2+^ (right panel), as well as in the presence of molar excess of the indicated GST-Kv7.2-CaMBD fusion proteins (200 nM). Note the difference in the fluorescence emission axis, as well as the shift in the peak emission to the left in the presence of Ca^2+^. The emission spectra of D-CaM alone and in the presence of I340E or S511D were indistinguishable. ***B.*** Relative fluorescence increase of D-CaM. Bars represent the mean ± SEM of the percentage increase in fluorescence emission (n≥4). ***C.*** Co-immunoprecipitation of full-length Myc-tagged Kv7.2 and CaM. Proteins from HEK293T cells expressing the constructs indicated were immunoprecipitated with the anti-Myc antibody, separated by SDS-PAGE and analyzed in Western blots probed with anti-Myc or anti-CaM antibodies (n = 4). ***D.*** Co-immunoprecipitation of N-terminal HA-tagged Kv7.3 assembled with N-terminal Myc-tagged Kv7.2. Western blots were probed with anti-Myc, anti-HA or anti-CaM antibodies (n = 4).

Using the yeast two-hybrid system we previously found that introducing a phosphomimetic mutation in helix B at position 511 precluded CaM binding to the Kv7.2 CaM binding domain [3]. To further corroborate this result, we have studied the interaction of dansyl-calmodulin (D-CaM) with the purified CaM-binding site (CaMBD) carrying this mutation. The results were compared to those obtained with the I340E mutant, a mutation that disrupt CaM binding, preventing surface expression [15]. D-CaM fluorescence increases in response to conformational changes upon binding to its target and we have shown that this increase in fluorescence is a robust predictor of the impact of several mutations in the CaM-binding site on Kv7.2 surface expression [14]. Whereas the interaction with CaMBD-WT caused a ∼100% increase in fluorescence, there was almost no change in the presence of molar excess of CaMBD-S511D, either in the presence or absence of Ca^2+^ (Fig. 1A, B). We also confirmed that the full-length S511D mutant channel does not associate with CaM, whereas mutating Ser 511 to Ala did not preclude CaM binding (Fig. 1C). Finally, CaM was not detected when the Kv7.2 S511D mutant was co-expressed with Kv7.3 subunits (Fig. 1D). This result is intriguing, since homomeric Kv7.3 channels bind CaM [3], and suggests that mutations in Kv7.2 can interfere with CaM binding to the Kv7.3 binding site in heteromers. In summary, regarding CaM binding, the impact of the S511D and I340E mutations were indistinguishable. Hence, in conjunction with previous yeast two hybrid and co-IP studies of the CaM interaction [3], these data demonstrate that the S511D mutation impairs CaM binding to Kv7.2.

**Figure 2:**
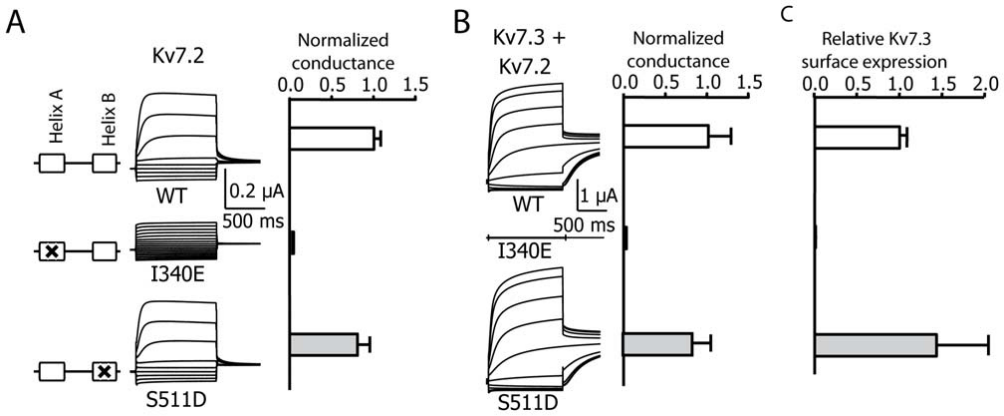
The CaM-binding incompetent S511D mutant is functional, whereas the CaM-binding incompetent I340E mutant is not. ***A.*** Left column, schematic illustration of the CaM binding region. Boxes represent helices A and B. Middle column, representative current traces from *Xenopus* oocytes expressing homomeric Kv7.2 WT, I340E or S511D channels. Currents were elicited by 800 ms jumps to potentials between -120 and +50 mV from a holding potential of -50 mV. Tail currents were measured at -20 mV. Right column**,** the difference in amplitude of the relaxation measured at -20 mV after a pulse to -120 mV and to +50 mV was measured. Bars show the mean relaxation amplitudes measured at -20 mV for oocytes expressing the indicated homomeric channels (n≥10). The difference in current between WT and S511D was not significant (Unpaired Student's *t* test). ***B.*** Representative current recordings from *Xenopus* oocytes injected in a 1∶1 ratio of cRNAs for Kv7.3, and Kv7.2, Kv7.2-I340E or Kv7.2-S511D. Right column, normalized average conductance. Bars represent the mean ± SEM of the normalized conductance of Kv7.2/3 heteromeric channels (n≥5). ***C.*** Normalized surface expression of Kv7.3 subunits tagged with HA in the extracellular S1-S2 loop after co-expression with Kv7.2, Kv7.2-I340E or Kv7.2-S511D. *Xenopus* oocytes were injected with a 1∶1 ratio of cRNAs for Kv7.3 and Kv7.2 (n = 12, two batches). The background of uninjected oocytes was subtracted and the values given are the means (± SEM) normalized to the values obtained from WT-Kv7.2/3 channels from the same batch. The difference in surface expression was not statistically significant (Unpaired Student's *t* test).

Contrary to previous proposals [10], we found that constitutive tethering of CaM to Kv7 channels is not a requirement for channel function. In contrast to the helix A I340E CaM binding mutant, we found no difference in the current recorded from *Xenopus* oocytes expressing either homomeric full length WT or S511D channels (Fig. 2A). Although the fact that homomeric Kv7.2 channels are functional implies that they reach the plasma membrane, the impact of this mutation on surface expression could not be addressed as surface expression of homomeric Kv7.2 channels is barely detectable [18]. However, surface expression can be measured when combined with Kv7.3 subunits [18]; [19]. Indeed, when we studied the impact of this mutation in Kv7.2/3 heteromers, currents were potentiated when the S511D mutant subunits were co-expressed with Kv7.3 subunits (Fig. 2B). The current levels correlated with channel surface expression (Fig. 2C) even though no CaM binding to Kv7.2-S511D/Kv7.3 heteromers could be detected (Fig. 1D). Thus, full-length WT and S511D channels were functionally indistinguishable.

**Figure 3:**
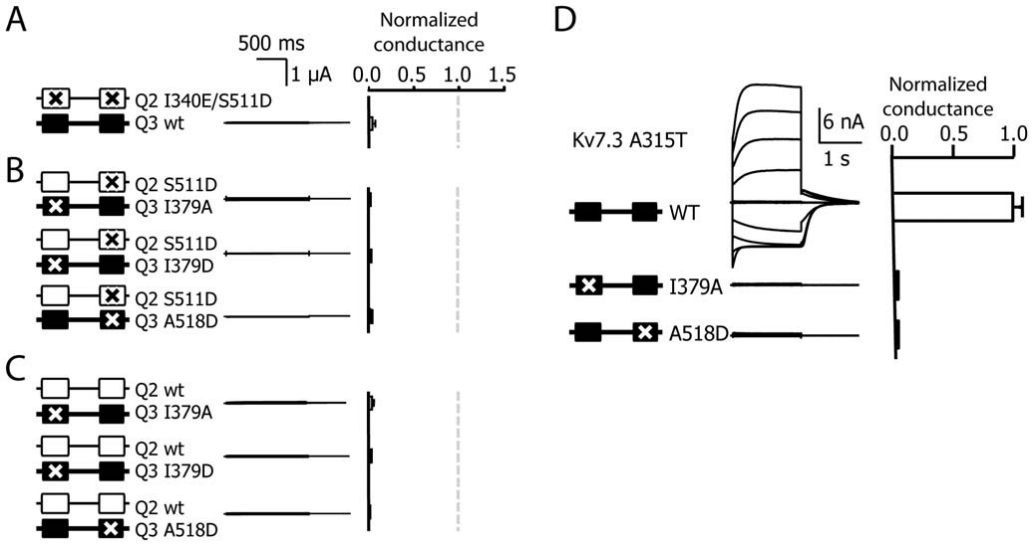
The S511D mutation did not restore the function of other CaM binding perturbing mutations. Left column, schematic illustration of the CaM binding region as in Fig. 2. Kv7.2 subunits are represented by white boxes and Kv7.3 subunits by black boxes. Crosses indicate point mutations known to prevent CaM binding to Kv7.2 introduced at equivalent position in Kv7.2 or Kv7.3. Middle column, representative current recordings from *Xenopus* oocytes injected in a 1∶1 ratio of cRNAs for Kv7.3, Kv7.2, or the different mutants studied. Right column, normalized average conductance. Bars represent the mean ± SEM of the normalized conductance mutant Kv7.2/3 channels (n≥4). The dotted line is the reference normalized conductance from K7.2/3. ***A.*** The double mutant I340E/S511D Kv7.2 co-expressed with WT Kv7.3 was not functional. ***B.*** Introduction of CaM binding perturbing mutations in helix A or helix B of Kv7.3 lead to non-functional channels. Left, schematic representation of the mutants analyzed. Middle column, representative current traces of whole-cell patch-clamp recordings from HEK293T expressing homomeric Kv7.3^T^ WT, I379A or A518D. The pore A315T mutation was introduced to boost expression [19]-[21]. ***C and D.*** Kv7.2 S511D or WT did not restore the function of Kv7.3 subunits carrying mutations in helix A or helix B. Currents were elicited with 800 ms jumps to potentials between -100 and +60 mV from a holding potential of -20 mV. Tail currents were measured at -20 mV. Right column, the difference in the amplitude of the relaxation measured at -20 mV after a pulse to -100 mV and +60 mV was measured (n≥8).

Mutations located in helix A of Kv7.2 or on either helix of Kv7.3 that perturb CaM binding had a dominant negative effect on function. In combination with the I340E mutation in helix A that abolishes CaM binding to Kv7.2, the S511D mutation resulted in non-functional channels, even though the double mutant was co-expressed with Kv7.3 subunits to facilitate its functional detection (Fig. 3A). In addition, when the influence of the corresponding mutations in helix A or helix B of Kv7.3 was assessed we found that the channels were not functional. Since Kv7.3 homomeric channels produce very small currents, we boosted surface expression by including the A315T pore mutation [19]-[21]. Even under these favorable conditions, no functional channels were produced for the helix A or B Kv7.3 mutants (Fig. 3B). The lack of function caused by the mutations in Kv7.3 persisted when combined with Kv7.2 (Fig. 3C and D). Thus, the consequence of a phosphomimetic mutation in helix B depends on the particular Kv7 subunit and it is not compatible with the function of Kv7.3 channels.

**Figure 4:**
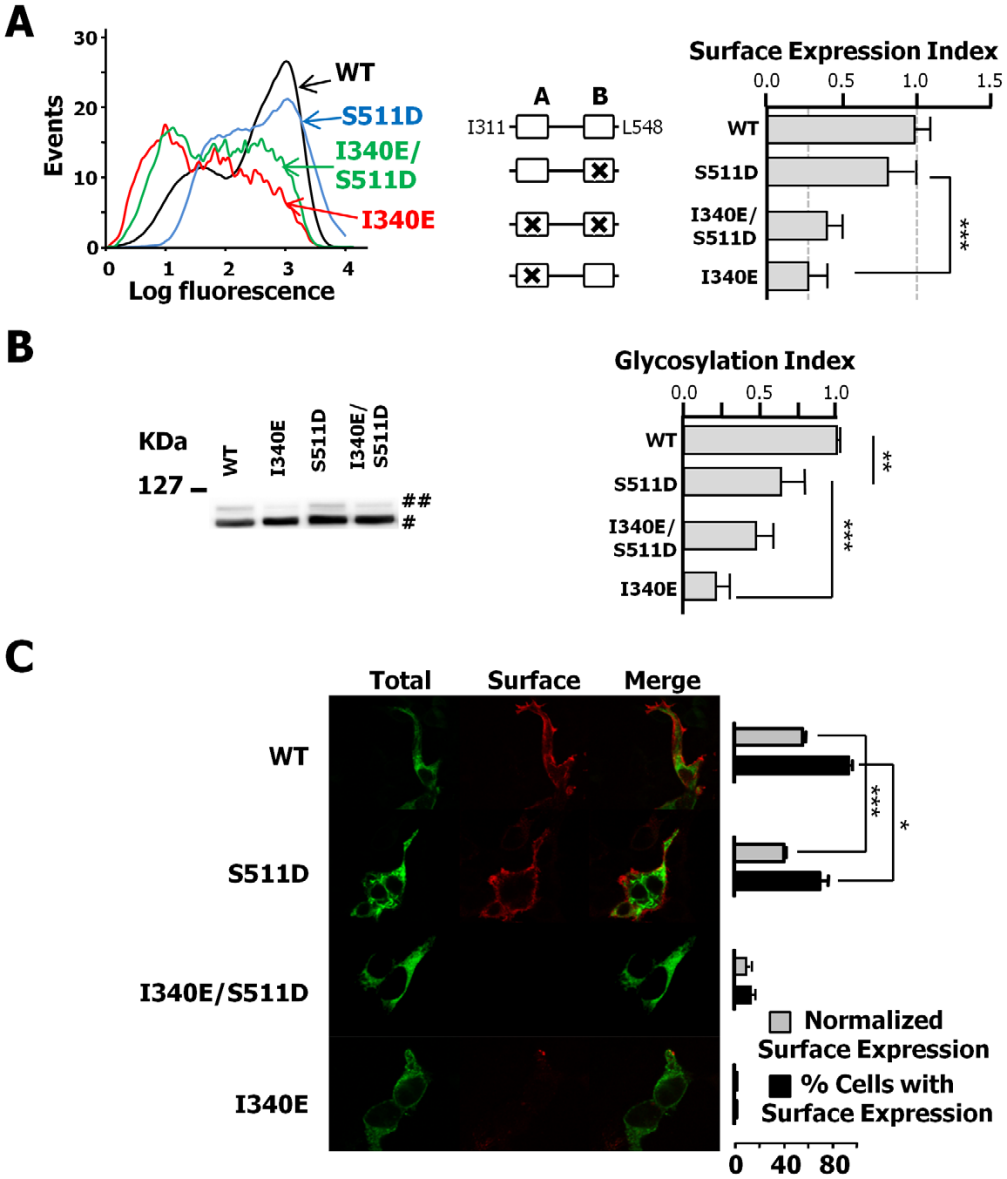
The double mutation I340E/S511D impairs plasma membrane expression. ***A. Left***, flow cytometry histogram of cell surface staining. ***Center***, schematic representation of the AB mutants analyzed. ***Right***, the normalized sum of the product of the number of events and fluorescence intensity from the flow cytometry histogram distribution is plotted as surface expression index. Dotted lines are the mean values of AB-I340E and AB-WT. There was no significant difference in the surface expression index of the S511D and WT construct, but the index was significantly different to that of I340E (n = 4). ***B.*** The glycosylation of the I340E/S511D mutant was larger than that of the I340E and not significantly different to that of the S511D mutant. Western blot probed with an anti-GFP antibody of HEK293T cell extracts expressing Tac-AB constructs carrying the indicated mutations. The bands represent immature (#) and mature (##) forms of the Tac chimeras. ***Right***, averaged ratio of the optical density (mature OD/(immature OD + mature OD)) normalized to the Tac-AB ratio (n≥3). ***, significance at *P*≤0.001, ** *P*≤0.01, paired Student's *t* test. ***C***. Confocal images of HEK293T cells transfected with WT and mutant channels tagged with an HA extracellular epitope and mCFP. A deletion between helix A and B was introduced to boost surface expression (see text). HEK293T cells transfected with the indicated cDNAs were surface stained with anti-HA antibodies. The proportion of the cells with surface staining was determined in > 40 CFP positive cells for each construct in three independent experiments. Grey bars represent mean ± SEM of the fluorescence intensities per unit area obtained for surface (red channel) divided by total fluorescence. Fewer cells expressed S511D at the surface (black bars), and in those cells, the average surface expression was reduced (grey bars). * *P*≤0.05; *** *P*≤0.001; unpaired Student's *t* test.

Two scenarios could account for the lack of function of the Kv7.2 double mutant: either the mutant channel does not reach the plasma membrane, or, if it does, the helix A mutation prevents function by an unknown mechanism. To distinguish between these two alternatives, and given the technical difficulties to detect homomeric Kv7.2 channels at the plasma membrane, we studied the influence of those mutations on traffic using a trafficking reporter protein (Tac). Tac (interleukin-2 receptor α subunit) is a single transmembrane glycosylated protein that has been used to identify determinants of surface expression [22], and we previously showed that the CaM-binding site controls trafficking when transferred to the Tac protein [15]. As expected, introduction of the helix A mutation I340E almost completely suppressed surface expression (Fig. 4), confirming the role of CaM in trafficking [14]; [15]. In addition, the glycosylation pattern, that is an indicator of passage to the Golgi apparatus, revealed a reduction on the intensity of the high molecular weight band, as previously reported and consistent with the proposed role of CaM binding for ER exit [14]; [15].

The trafficking parameters obtained for the S511D mutant and the double mutant were intermediate between those of I340E and WT. There was no significant difference between the surface expression of Tac-AB-S511D and Tac-AB-WT. Similarly, the difference in surface expression between Tac-AB-I340E and Tac-AB-I340E/S511D was not statistically significant. In addition, the extent of glycosylation revealed a graded pattern. Based on the intensity of the high molecular band, the rank was WT > S511D > S511D/I340E > I340E. Thus, it appears that the helix B mutation partially counteracts the impact of the helix A mutation. However, in some instances the results obtained using chimeric proteins are no consistent with those obtained using the full-length ion channel [23]. Fortunately, during the course of this study, we found that surface expression of Kv7.2 could be increased by removing the loop connecting helix A and helix B (Y372-K493, P.A., in preparation), facilitating their detection. This permitted us to circumvent the technical limitations mentioned above, allowing evaluating the impact of the mutations in homomeric Kv7.2 channels.

Consistent with the results obtained using the Tac reporter, while I340E Kv7.2 CaM-binding deficient mutant channels did not reach the cell surface, S511D Kv7.2 mutant channels did (Fig. 4C). In combination with the helix A-B loop deletion the S511D mutant yielded current levels indistinguishable from WT channels (Fig. S1). Nevertheless, its surface expression was reduced when compared to WT subunits (Fig. 4C). As a result, we conclude that the restoration of surface expression endowed by the S511D mutation is partial, which is consistent with the reduction in glycosylation observed with the Tac reporter (Fig. 4). Thus, there must be a compensatory mechanism that allows the S511D mutant to yield similar current levels to the WT, albeit with fewer channels at the plasma membrane. In contrast, the double mutant did not reach the surface, explaining its lack of function and revealing that the helix A mutation I340E exerts a negative dominant effect on traffic.

**Figure 5:**
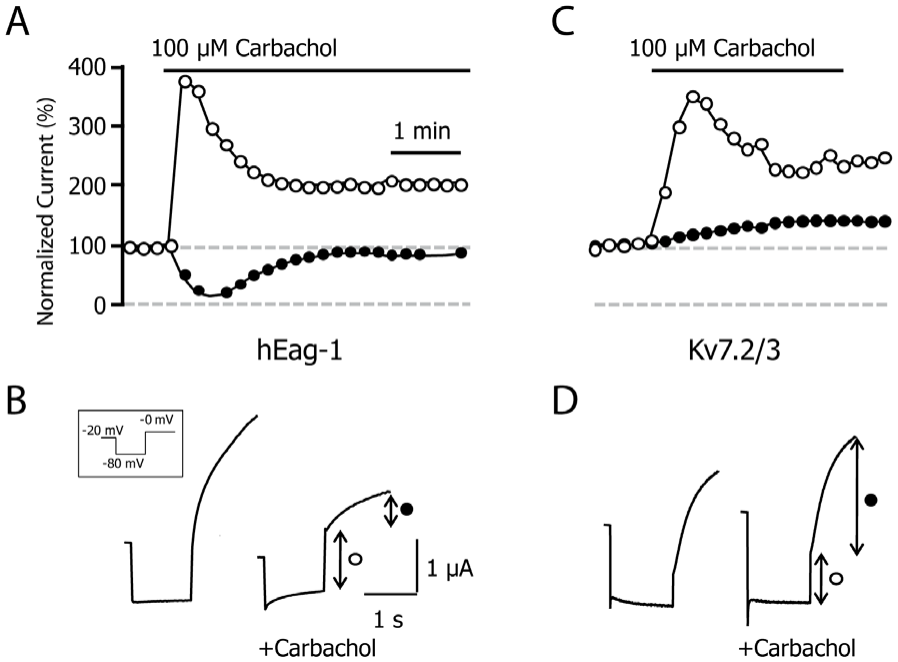
Activation of Ca^2+^-CaM does not inhibit Kv7.2/3 in *Xenopus* oocytes. ***A.*** Representative time-course of M_1_-muscarinic receptor activation by carbachol when expressed in oocytes co-expressing Ca^2+^-CaM regulated hEag-1 potassium channels (n = 4). The normalized instantaneous current from −80 mV to −0 mV is plotted as white circles and corresponds to the activation of the endogenous Ca^2+^-dependent Cl^-^ current. The size of the post-pulse relaxation at 0 mV is plotted as black circles and corresponds to the opening of hEag-1 channels. The activation of the Cl^-^ current demonstrated that intracellular Ca^2+^ increased, and the inhibition of the hEag1 currents demonstrated that oocytes support Ca^2+^-CaM-mediated inhibition of potassium channels. ***B.*** Representative traces from A before and during the application of 100 µM carbachol. ***C.*** Representative time-course of M_1_-muscarinic receptor activation when expressed in oocytes co-expressing Kv7.2/3 potassium channels (n = 6). Same layout as in A. The size of the post-pulse relaxation at 0 mV is plotted as black circles and corresponds to the opening of Kv7.2/3 channels. ***D.*** Representative traces from C before and during the application of 100 µM carbachol.

CaM exerts multiple effects on Kv7.2 channels and, in addition to regulating ER exit, it also mediates Ca^2+^-dependent inhibition of the M-current [6]. In order to assess the impact of the S511D mutation on CaM mediated regulation, we took advantage of the well known transient increase in intracellular Ca^2+^ in response to the activation of phospholipase C coupled receptors in *Xenopus* oocytes. To demonstrate the activation of CaM, the activation of M_1_-muscarinic receptors on co-expressed hEag-1 potassium currents was monitored (which have an IC_50_ for Ca^2+^ of 106 nM, reaching maximal inhibition at > 5 µM Ca^2+^), provoking the inhibition expected [24] in conjunction with the activation of the endogenous chloride current (Fig. 5A and B).

Ca^2+^-CaM activation did not lead to Kv7.2/3 current inhibition, but rather a small increase in current was consistently observed (Fig. 5C and D). Plasma membrane reduction of PIP_2_ levels is thought to be the primary mechanism for M-current inhibition by muscarinic receptors [25]. On the other hand, Ca^2+^-mobilizing hormones may stimulate PIP_2_ synthesis in addition to promoting its breakdown [26]. In *Xenopus* oocytes, synthesis may overcome breakdown in response to muscarinic activation. Indeed, little or no inhibition of Kv7.1 or Kv7.2 channels has been observed when M_1_-muscarinic receptors are activated in *Xenopus* oocytes [27]; [28], whereas a small reduction has been observed for Kv7.2/3 heteromers in oocytes treated with thapsigargin to deplete intracellular calcium stores [28].

**Figure 6:**
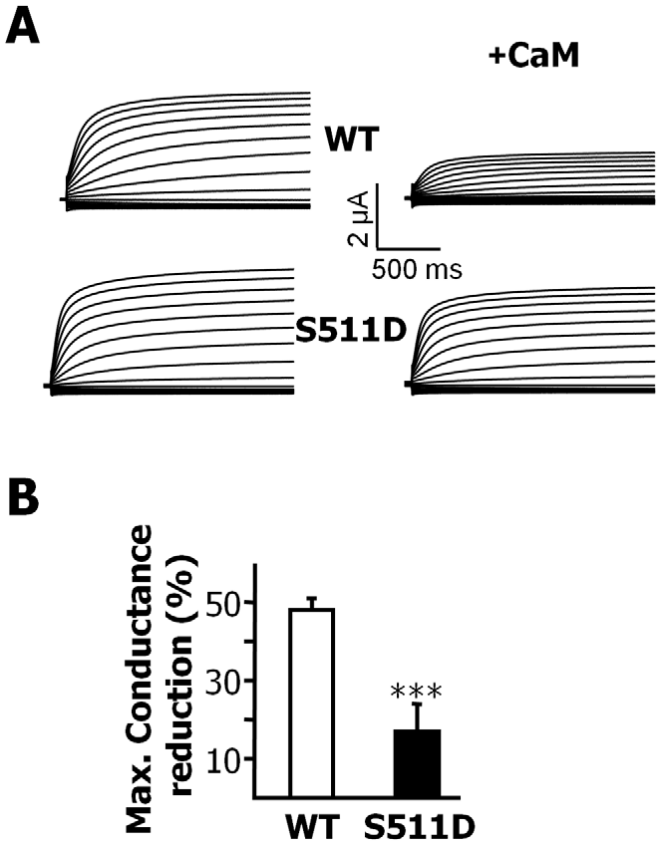
Effect of CaM overexpression on maximal conductance. Bars represent the mean relative reduction in maximal conductance co-expressing Kv7.3 with either WT or S511D subunits after overexpression of CaM in *Xenopus* oocytes. The maximal conductance was obtained after fitting a Boltzmann distribution to the current-voltage relationships from tail currents measured at −20 mV. Currents were elicited by 800 ms jumps to potentials between −120 and +50 mV from a holding potential of -50 mV. There was a significant difference between the two groups (*** *P*<0.0001, unpaired Student's *t* test: n≥6; two batches of oocytes).

CaM overexpression had a weaker effect on S511D mutant channels than on WT channels. Under resting Ca^2+^ conditions, the reduction in the M-current produced by CaM can be demonstrated by overexpressing CaM, and the extent of this reduction correlates with the sensitivity of different Kv7 subunits to Ca^2+^-CaM mediated modulation [6]; [7]. We found that the overexpression of CaM led to a significant reduction in maximal conductance in oocytes, replicating the effect observed in CHO cells [6]; [7]. By contrast, the effect of CaM on the S511D mutant (co-expressed with Kv7.3) was significantly reduced (Fig. 6), which is consistent with the idea that helix B directly funnels the effects of CaM on Kv7 channel function in addition to controlling trafficking.

## Discussion

CaM interacts with a wide range of proteins and regulates a large variety of cellular functions. Among these, CaM has been shown to regulate the trafficking of membrane proteins at different levels, including assembly, ER exit and endocytosis [12]; [13]; [15]; [29]-[33]. This function is critical for ion channels whose number and composition at the cell surface determines how cells respond to particular stimuli. The data available indicate that CaM binding is a strict requirement for Kv7.2 trafficking: there is a strong correlation between CaM binding and surface expression, decreasing CaM levels leads to ER retention and reduced hippocampal M-current function, and CaM overexpression promotes ER exit [14]; [15]; [34].

Similar to SK channels, that are intrinsically associated with CaM [9], Kv7 channels bind CaM even in the absence of Ca^2+^ [3]; [10]. The concept of constitutive tethering to an ion channel is clearly illustrated for SK channels because harsh treatments, such as altering pH or ionic strength by two orders of magnitude or more are ineffective at stripping CaM from inside out patches [9]. On the basis that Kv7.2 mutants deficient in CaM binding were unable to generate detectable currents, it was proposed that CaM is an auxiliary subunit of M-channels [10]. The finding that the S511D mutant does not bind CaM yet traffics to the membrane is clearly surprising and demonstrates that constitutive tethering of CaM is not a requirement for M-channel function.

Our working hypothesis is that CaM conceals a retention/retrieval signal, and that S511D causes two effects that counterbalance one another: it impedes constitutive CaM binding but it does not lead to intracellular channel retention because the appropriate trafficking signal is neutralized. We hypothesize that this mutant causes a reorientation of the region that represents a trafficking determinant, such that it is no longer exposed. Alternatively, the S511D mutation mimics CaM binding by stabilizing a conformation of the protein similar to that adopted when CaM is bound, making CaM binding superfluous.

If CaM binding is unnecessary, any additional mutation that prevents CaM binding should be inconsequential. Therefore, the lack of function of the helix A and helix B double mutant is puzzling. Our data shows that the double mutant does not reach the membrane and, therefore is not functional. If CaM occludes a retention signal that can be neutralized by the S511D mutation, additional mutations in the CaM binding site should not prevent surface expression. Thus, the process is undoubtedly more complicated than the simple masking hypothesis that is guiding our work. Perhaps there are additional trafficking signals within other parts of the CaM binding site. However, this would not seem to be sufficient, because those signals should then have caused intracellular retention of the S511D mutant, since it does not bind CaM. One property that should be considered is that helix A alone can interact with CaM *in vitro* [6]; [10]. Therefore, the S511D mutant channel contains a cryptic CaM binding site constituted by the intact helix A. Perhaps the CaM interaction with helix A is so transient in this mutant that it cannot be detected by the methods available, but this transient interaction is required for trafficking.

While we expected that the introduction of the mutation equivalent to S511D in Kv7.3 subunits would lead to functional channels, this was not the case, and the Kv7.3 A518D mutant was not even functional after interventions known to boost surface expression, such as co-expression with Kv7.2 or introduction of the A315T pore mutation. We should consider the possibility that the role of CaM binding may be distinct in the different Kv7 isoforms. For instance, there is evidence that CaM binding is required for Kv7.1 subunit assembly [12]; [13], whereas our data suggest that this is not the case for Kv7.2 [15].

We also found that the S511D mutation only partially restores trafficking, yet the currents produced are indistinguishable. This may be due to an increase in single channel conductance or to an increase in the probability of the channel being open (*P_o_*). It is unlikely that the single channel conductance is affected by this mutation, since this property depends on the pore domain. Instead, we propose that the *P_o_* increases in the CaM-independent mutant channel. Under resting intracellular Ca^2+^ conditions, CaM causes a reduction in current [6]; [7]. Thus, removing this tonic inhibition should increase the maximal current, as observed for the S511D mutant, compensating for the reduction in channel trafficking.

The identification of a mutation that prevents CaM binding but that still yields functional channels provides us with a useful tool to study Ca^2+^-CaM-dependent regulation [6]; [7]. Unexpectedly, Kv7.2/3 channels expressed in oocytes do not respond to increases in intracellular Ca^2+^ triggered by the activation of heterologously expressed M_1_-muscarinic receptors, whereas the same paradigm does support Ca^2+^-CaM-mediated suppression of hEag-1 potassium currents. This was surprising, given that the affinity of the Kv7 CaM-binding site for Ca^2+^-CaM is similar or even higher to that reported for the hEag-1 CaM-binding site (K_D_ ∼25 nM for Kv7.2 and Kv7.3 (A.A. in preparation) *vs* >100 nM for the hEag-1 CaM-binding site; [35]).

Reductions in plasma membrane PIP_2_ levels are thought to be the primary mechanism for M-current inhibition by muscarinic receptors [25]. It was speculated that CaM may act by decreasing the affinity of Kv7 for PIP_2_ [36]. Accordingly, increasing PIP_2_ levels could prevent CaM mediated inhibition and enhance Kv7 current. This would be consistent with the small increase in current we observed in oocytes after M_1_-muscarinic receptor activation. It has been suggested that Ca^2+^-CaM inhibition may be mediated by an additional low affinity CaM site [6], which would explain the lack of Ca^2+^-dependent inhibition in oocytes. In conclusion, the lack of Kv7 inhibition in oocytes imposes severe restrictions on the plausible mechanisms for Ca^2+^-CaM mediated M-current inhibition, and suggest that it requires the participation of additional mediators (such as PIP_2_).

Of the Kv7 subunits, only Kv7.1 has a consensus PKC site (S/T)*X*(R/K) at the equivalent position to Kv7.2 S511, and each known Kv7.2 orthologue in fishes and tetrapods maintains an equivalent PKC site. Interestingly, a consensus PKC site displaced three positions downstream can be found in Kv7.4 and thus, PKC activation can potentially regulate Kv7.2, Kv7.1 and K7.4 channel trafficking. However, introduction of the equivalent phosphomimetic mutation in Kv7.3 yielded non-functional channels, presumably because the channels did not reach the plasma membrane. Therefore, the effect of modifying helix B in other Kv7 subunits is difficult to predict, since it appears to be context-dependent.

PKC phosphorylation inhibits mGluR5 binding to CaM, decreasing mGluR5 surface expression [32]. By contrast, we would expect Kv7.2 surface expression to be only partially reduced by phosphorylating S511, leading to little change in the total current owing to removal of tonic CaM inhibition. However, there are multiple PKC sites in Kv7.2 that may override the mechanisms described here. Indeed, activation of PKC was previously shown to cause phosphorylation of another PKC site in helix B and inhibition of the current [37].

In summary, we have found that constitutive CaM binding is not a requirement for Kv7 channel function. Further experiments will be necessary to understand the implications of CaM binding on the different Kv7 subunits, and to fully understand how CaM controls traffic of Kv7.2 subunits.

## Materials and Methods

### Molecular biology

The human Kv7.2 (Y15065) and Kv7.3 (NM004519) cDNAs were provided by T. Jentsch (Leibniz-Institut für Molekulare Pharmakologie, Berlin, Germany) and cloned into pCDNA3.1 (Invitrogen). For immunoprecipitation experiments, the Kv7.2 subunit was tagged at the N terminus with a tandem repeat of five Myc epitopes (MEQKLISEEDLN), while the Kv7.3 subunit was tagged with a tandem repeat of two HA epitopes (YPYDVPDYA), and then both were sub-cloned into pCAGGS. The electrophysiological properties of N-tagged Kv7.2 and Kv7.3 constructs were indistinguishable from those of the non-tagged equivalents when recorded in *Xenopus* oocytes. The Tac-mCFP construct was generated using the Tac receptor provided by the group of R.J. Wenthold (NIDCD, National Institutes of Health, Bethesda, MD, USA), which was cloned into a modified version of the pEGFP expression vector (Clontech) where the eGFP had been replaced by mCFP. Different fragments of Kv7.2 were inserted in between Tac and mCFP. The cDNA encoding rat CaM was provided by the group of J.P. Adelman (Vollum Institute, Portland, OR, USA).

### Cell culture and transfection

HEK293T (HEK 293T/17, ATCC, CRL-11268) and CHO cells (CHO-K1, ATTC, CCL-61) were maintained at 37°C and 5% CO_2_ in Dulbecco's Modified Eagle's Medium (DMEM, Sigma-Aldrich), supplemented with non-essential amino acids (Sigma) and 10% FBS (Gibco). Cells were transiently transfected with cDNAs using a calcium phosphate protocol.

### Immunoprecipitation

Thirty six hours after transfection, one confluent T-75 flask of HEK293T cells was solubilized for 30 min at 4°C in IP buffer, containing (mM): 50 Tris-HCl, 150 NaCl, 1% Triton X-100, 2 EDTA, 5 EGTA (pH 7.5), and protease inhibitors (1X Complete; Roche Applied Science). The nuclei were recovered by centrifugation at 500 *g* for 3 min, and the insoluble material was removed after centrifugation at 11,000 *g* for 20 min. before the lysates were pre-cleared for 1 h at 4°C with 40 µl of equilibrated protein A-Sepharose beads (Sigma P3391). Anti-Myc antibodies were immobilized overnight at 4°C with 40 µl of equilibrated protein A beads and washed twice with immunoprecipitation buffer. Precleared lysates were incubated with protein A-anti-Myc for 4 h at 4°C, and after four washes with immunoprecipitation buffer, the immunoprecipitated proteins were released by heating at 90°C 5 min in SDS sample buffer.

### Antibodies

The following primary monoclonal antibodies were used: mouse anti-Myc (1∶1,000; 9E10, Sigma-Aldrich); rat anti-HA (1∶1,000; 3F10; Roche Applied Science); mouse anti-CaM (1∶2,000; Millipore); mouse anti-GFP (1∶2,000; clones 7.1 and 13.1; Roche Applied Science) and mouse anti-Tac (1∶1,000; BD Bioscience). Anti-mouse IgG peroxidase-coupled (1∶5,000; Bio-Rad Laboratories), and fluorescent secondary goat anti-mouse AlexaFluor 488 (1∶1,000; Invitrogen) secondary antibodies used were used.

### Electrophysiological measurements

Standard two-electrode voltage clamp procedures were employed to record currents from oocytes obtained from female *Xenopus* [19]. Currents were elicited with an 800 ms depolarizing step from −120 to +50 mV every 20 s, and tail currents were measured at −20 mV. HEK393T cells were used for whole-cell patch recordings, obtained at RT (21–25°C) one day after transfection using lipofectamine 2000 (Invitrogen). Cells were bath perfused with the following solution (mM): 136 NaCl, 4 KCl, 1.8 CaCl_2_, 1 MgCl_2_, 10 HEPES (Na), 10 D-glucose, adjusted to pH 7.4 with NaOH. Pipettes were pulled from borosilicate glass capillaries (Sutter Instruments, USA) and they had resistances of 1–3 MΩ. Membrane currents were measured using an Axopatch 200B amplifier (Axon Instruments, USA) with pipette and membrane capacitance cancellation, and they were sampled at 1 ms and filtered at 300 Hz. Pipettes were filled with an internal solution containing (mM): 125 KCl, 10 HEPES, 5 MgCl_2_, 5 EGTA, 5 Na_2_ATP, adjusted to pH 7.2 with KOH. The access resistance was typically 10 MΩ. The amplitude of the Kv7 current was defined as the peak difference of the current relaxation measured at −20 mV after 800 ms prepulses to −100 mV (all channels closed) and to +60 mV (all channels opened). Where indicated, Chinese hamster ovary (CHO) were used for whole-cell perforated patch using an EPC-8 amplifier (HEKA Instruments, Germany). Pipettes were filled with an internal solution containing (mM): 80 K acetate, 30 KCl, 40 HEPES, 3 MgCl_2_, 3 EGTA and 1 CaCl_2_, adjusted to pH 7.4 with KOH. Amphotericin B (Sigma) at 480 µg/ml was used to perforate the patch, prepared as a 60 mg/ml stock solution in DMSO. In these experiments, the access resistance was typically 10 MΩ 5–10 min after seal formation. The data were acquired and analyzed using pCLAMP software, normalized in Excel (Microsoft Corp., Madrid, Spain) and plotted in SigmaPlot (SPSS Corp., Madrid, Spain), and the data are shown as the mean ± SEM (*n* indicates the number of samples). The differences between the means were evaluated using the unpaired Student's *t* test and values of *P*≤0.05 were considered significant.

### Surface expression

A Kv7.3 subunit tagged with an HA epitope in the extracellular loop connecting the S1 and S2 transmembrane domains was used in chemiluminescent assays of individual oocytes [18]; [19]. The background signal from uninjected oocytes was subtracted (background represented <10% of the maximal signal).

### Flow cytometry

HEK293T cells grown in 35 mm dishes were transiently transfected with Tac-Kv7.2-mCFP by the calcium phosphate method. After 36 h, the cells were detached and disaggregated using a buffered solution of trypsin-EDTA and dissociation buffer (1∶2, Gibco), they were transferred to 1.5 mL Eppendorf tubes, washed and resuspended in 700 µL PBS. Cells were fixed for 20 min at RT with 3% paraformaldehyde (Fluka) and washed with PBS. After pre-incubation with 5% BSA (Sigma) for 30 min, the cells were labeled for 1 h at RT with an antibody recognizing a Tac extracellular epitope. They were then washed three times with PBS, blocked for 15 min with 5% BSA and incubated for 1 h at RT with an AlexaFluor 488 conjugated anti-mouse IgG secondary antibody (Invitrogen). Cells were washed four times and resuspended in PBS before they were analyzed on a FACSCalibur flow cytometer (Becton Dickinson Bioscience). Data were collected from at least 10,000 healthy cells with emission intensities above the background level that was determined using untransfected HEK293T cells. Histograms normalized to 10,000 events were visualized using WinMDI 2.9 software, analyzed in Excel and represented using SigmaPlot. The sum of the product of the number of events and fluorescence intensity was used as an index of surface expression.

### Confocal microscopy

Confocal microscopy was used to analyze surface expression of full-length channels. The extracellular loop of Kv7.2 that connects transmembrane domains S1 and S2 was tagged with two HA epitopes. To increase accessibility, the epitope was flanked with fragments from the extracellular D1-D2 loop of the ClC-5 chloride channel [18]. This insertion changes the sequence between transmembrane domains S1 and S2 to ^115^ KEYEKSSEGSEH**YPYDVPDYA**G**YPYDVPDYA**VTFEERDKCPEWNA^126^. The loop connecting helix A and helix B (Y372-K493) was removed to increase surface expression (P.A., in preparation). HEK293T were grown in poly-L-Lysine coated coverslips (treated for 30 min with 1 mg/mL poly-L-Lysine, Sigma) were washed with PBS 36 h after calcium phosphate transfection. The sample was fixed with freshly diluted 3% paraformaldehyde in PBS for 20 min, washed three times with PBS and then pre-incubated for 30 min with 5% BSA. The primary antibody (anti-HA diluted 1∶1,000 in blocking solution) was then added for 1 h, after which the cells were washed three times in PBS, blocked with 5% BSA for 20 min and exposed for 1 h to the secondary antibody (AlexaFluor 594-conjugated goat anti-rat IgG, Invitrogen diluted 1∶1,000 in blocking solution). The cells were then washed three times in PBS and mounted with ProLong Antifade reagent (Invitrogen). Images from stained cells were captured using a 60X oil objective on a Nikon Eclipse TE2000-U fluorescence microscope equipped with a confocal module using a 30 µm pinhole. Image acquisition and analysis were performed blind, with no knowledge of the construct transfected. ImageJ software (Version WCIF, National Institutes of Health, USA) was used to quantify surface expression. Fluorophores were excited with a 437 nm laser (Radius, Coherent) for CFP, or a 560 nm laser line (Melles-Griot) for AlexaFluor 594. The emission filters used were BA 485/40 (Chroma) for CFP and BA593/40 (Nikon) for AlexaFluor 594.

### Recombinant protein production and fluorescence analysis

CaM dansylation and the purification of CaM and GST-fusion proteins for the Kv7.2 WT, I340E, S511D CaM-binding site (G310-L548) was carried out as described previously [14]. Dispersion of the samples was evaluated by dynamic light scattering using a Zetasizer Nano instrument (Malvern Instruments Ltd.) in order to exclude the presence of aggregates. The correlation function and the polydispersity index (less than 0.2) demonstrated that the proteins were monodispersed and no aggregates were found. Fluorescence emission spectra of D-CaM were recorded at RT using an Aminco Bowman series 2 fluorescence spectrophotometer (SLM Aminco). The exwavelength was 340 nm, and emissions were collected between 400 and 600 nm. In these experiments, 200 nM of each GST-fusion protein was added to a 100 µL cuvette containing 12.5 nM of D-CaM alone (in presence of EGTA 10 mM) or in the presence of excess Ca^2+^ (5.1 mM Ca^2+^ to give 2.0 µM free Ca^2+^) in fluorescence buffer (mM): 25 Tris-HCl, 120 KCl, 5 NaCl, 2 MgCl_2_ and 10 EGTA (pH 7.4).

## Supporting Information

Figure S1 ***A.*** Representative traces of whole-cell perforated patch-clamp recordings from CHO cells transfected with the cDNAs indicated that encode tagged subunits with an HA extracellular epitope and an intracellular mCFP tag. To boost expression, a deletion between the loop connecting helix A and helix B was introduced. Currents were elicited with 1,500 ms jumps to potentials between −100 and +80 mV from a pre-potential of +10 mV. Tail currents were measured at −30 mV. ***B.*** The difference in the amplitude of the relaxation measured at −30 mV after a pulse to −100 mV and +80 mV was measured. Bars show the mean current density measured at −30 mV for cells expressing the indicated channels (n≥17). The difference in current densities was not significant (Unpaired Student's *t* test).
(TIF)
