# Correction: Kv7 Channels Can Function without Constitutive Calmodulin Tethering

**DOI:** 10.1371/annotation/ba5c7e72-f4a7-46b1-875b-138bc98b83c3

**Published:** 2014-01-10

**Authors:** Juan Camilo Gómez-Posada, Paloma Aivar, Araitz Alberdi, Alessandro Alaimo, Ainhoa Etxeberría, Juncal Fernández-Orth, Teresa Zamalloa, Meritxell Roura-Ferrer, Patricia Villace, Pilar Areso, Oscar Casis, Alvaro Villarroel

Figure 3 is incorrect. Please see the correct Figure 3 here: http://plosone.org/corrections/pone.0025508.g003.cn.tif

In addition, there was an error in the legend for Figure 3B. The legend should read: 

B. Introduction of CaM binding perturbing mutations in helix A or helix B of Kv7.3 lead to non-functional channels. Left, schematic representation of the mutants analyzed. Middle column, representative current traces of whole-cell patch-clamp recordings from HEK293T expressing homomeric Kv7.3T WT, I379D or A518D. The pore A315T mutation was introduced to boost expression [19]-[21].

