# Supplementary figures and images for: Kv7 Channels Can Function without Constitutive Calmodulin Tethering
Source: PLoS One. 2011 Sep 28;6(9):e25508. doi: 10.1371/journal.pone.0025508 (PMC3182250; doi:10.1371/journal.pone.0025508)

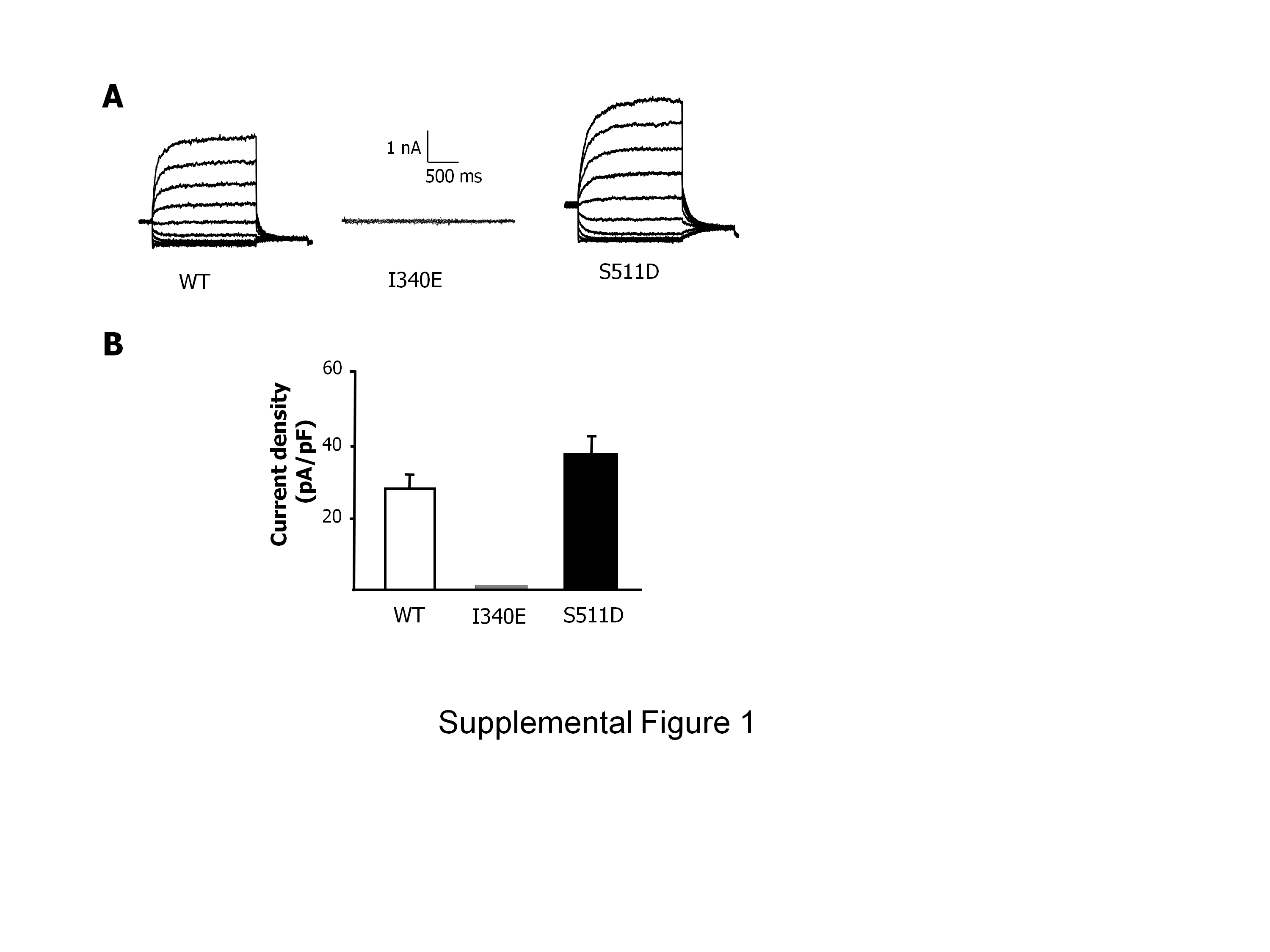

Supplement: Figure S1 — A. Representative traces of whole-cell perforated patch-clamp recordings from CHO cells transfected with the cDNAs indicated that encode tagged subunits with an HA extracellular epitope and an intracellular mCFP tag. To boost expression, a deletion between the loop connecting helix A and helix B was introduced. Currents were elicited with 1,500 ms jumps to potentials between −100 and +80 mV from a pre-potential of +10 mV. Tail currents were measured at −30 mV. B. The difference in the amplitude of the relaxation measured at −30 mV after a pulse to −100 mV and +80 mV was measured. Bars show the mean current density measured at −30 mV for cells expressing the indicated channels (n≥17). The difference in current densities was not significant (Unpaired Student's t test). (TIF) [file pone.0025508.s001.tif]
